# The Need for Integrative Computational Oncology: An Illustrated Example through MMP-Mediated Tissue Degradation

**DOI:** 10.3389/fonc.2013.00194

**Published:** 2013-07-26

**Authors:** Shannon M. Mumenthaler, Gianluca D’Antonio, Luigi Preziosi, Paul Macklin

**Affiliations:** ^1^Center for Applied Molecular Medicine, Keck School of Medicine, University of Southern California, Los Angeles, CA, USA; ^2^Dipartimento di Scienze Matematiche, Politecnico di Torino, Torino, Italia; ^3^Department of Biomedical Engineering, Viterbi School of Engineering, University of Southern California, Los Angeles, CA, USA

**Keywords:** matrix metalloproteinase, tissue degradation, integrative modeling, cancer, computational oncology

## Abstract

Physical oncology is a growing force in cancer research, and it is enhanced by integrative computational oncology: the fusion of novel experiments with mathematical and computational modeling. Computational models must be assessed with accurate numerical methods on correctly scaled tissues to avoid numerical artifacts that can cloud analysis. Simulation-driven analyses can only be validated by careful experiments. In this perspectives piece, we evaluate a current, widespread model of matrix metalloproteinase-driven tissue degradation during cancer invasion to illustrate that integrative computational oncology may not realize its fullest potential if either of these critical steps is neglected.

## Introduction

Physical oncology – the study of the physical biology of cancer, the development of new physical measurement platforms, and the use of mathematical and computational modeling to understand complex cancer systems – has emerged as an important force in cancer research (1). Key to this approach is integrative computational oncology: multidisciplinary teams of biologists, oncologists, physicists, engineers, and mathematicians working together to generate novel platforms, where modeling informs experiments, and experiments drive modeling. Mathematical modeling can describe and simplify complex systems, facilitating analysis. Accurate simulations assist the analysis of these systems, yielding observations that drive biological hypotheses. Experimental biology is necessary for validating and refining these hypotheses and advancing our understanding of cancer. This special issue discusses successful examples of applying integrative modeling to cancer-related questions. However, neglecting any of these key ingredients can be detrimental and may blind teams to subtle modeling flaws, potentially resulting in misleading model assessment, incorrect biological conclusions, or unverifiable predictions.

In this perspectives piece, we will look at a widely used mathematical model of tissue degradation by matrix metalloproteinases (MMPs) in order to illustrate (1) the need for evaluation of mathematical models by proper numerical techniques, applied to biologically relevant space and time scales, and (2) that even with proper numerical analysis, only experiments can truly validate mathematical model predictions and help choose among plausible explanations of model findings.

## MMP-Mediated Tissue Degradation

Progression from *in situ* carcinoma – where growth is constrained to a local site by a fully intact basement membrane (BM) – to invasive carcinoma requires disruption of the BM and penetration into the surrounding stroma. Once in the stroma, invading cancer cells often degrade and remodel the extracellular matrix (ECM) and later break through BM to enter blood vessels – a key step in metastasis. A quantitative understanding of proteolytic degradation of tissue is necessary in predicting (and disrupting!) cancer invasion and metastasis. It is currently unclear whether tissue degradation is primarily due to MMPs secreted by cancer cells or by stromal cells in response to tumor signaling. Quantitative modeling could help narrow down the possibilities to the most plausible models of stromal invasion, which can then be experimentally tested and validated.

Extracellular matrix is a 3-D cross-linked network of proteins and polysaccharides that provides structural support to cells; BM is a specialized form of ECM, although thinner (50–100 nm) and more dense (2, 3). ECM (including BM) can be degraded by MMPs secreted by tumor, stromal, and immune cells (4–6). MMPs are secreted in an inactive form that must be cleaved into an active form, and are further regulated by inhibitors of metalloproteinases. MMPs may be soluble and diffuse through tissue (e.g., MMP9), or membrane-bound (e.g., MT-MMP1) (6).

The most widely used tissue degradation models focus on soluble MMPs using reaction-diffusion equations [e.g., (7–9)], neglect inhibitors and promoters, and assume the MMP is immediately active. If *E* is the ECM density (or volume fraction) and *M* is the MMP concentration (both dimensionless), then

(1) $$\frac{\partial M}{\partial t}=\nabla \cdot (D\nabla M)+s(\text{X},\;t)-r_{M}EM-d_{M}M$$

(2) $$\frac{\partial E}{\partial t}=-r_{E}EM.$$

*D* is the diffusion constant, *s* is the source (tumor or other cells), *r_M_* and *r_E_* are reaction rates, and *d_M_* is the MMP decay rate. These standard reaction-diffusion equations are typically solved with finite differences on a Cartesian mesh [e.g., (7–9)]. The most clinically oriented BM degradation model we know of simulated BM as denser ECM on the same ECM computational mesh (10).

### Functional forms and parameter values

For this discussion, we assume *D* = *D*_0_ (1 − *E*) for a constant *D*_0_. We set *D*_0 _= 8 × 10^−9^ cm^2^/s (11), *r_M_* = *r_E_* = 1/200 s^−1^ (11), and *d_M_* = 5 × 10^−5^ s^−1^ (12). This gives an (ECM-dependent) reaction-diffusion length scale *L* of $\sqrt{D_{0}\left(1-E\right)∕\left(r_{M}E+d_{M}\right)}$ ~10 μm (“ ~”denotes “on the order of”) for 0 ≤ *E*  ≤ 0.90. This matches our biophysical expectations: MMPs are relatively large macromolecules diffusing through a tortuous ECM structure, so the length scale should be significantly smaller than for oxygen (typically ~100 μm). We initially set the *E* = 0.85.

### Model evaluation requires good numerical techniques

Accurate numerical solution (and hence proper evaluation) of the model and its ~10 μm length scale requires an ~1 μm mesh size. To date, most published work has used small diffusion constants on relatively large 10–20 μm meshes [e.g., (7, 8)]. We solve Eqs 1 and 2 on a 1 μm mesh with standard centered finite differences, using the ghost fluid method to implement boundary conditions wherever the computational stencil intersects the BM (zero flux, or Neumann condition) or a cell boundary (constant, or Dirichlet condition for secreting cells; Neumann condition for non-secreting cells) (13–15). We describe the BM position as in D’Antonio et al. (16). Tumor cell sizes are set to the values in Macklin et al. (17).

### Current models predict rapid “tunneling” through ECM

**Figure 1:**
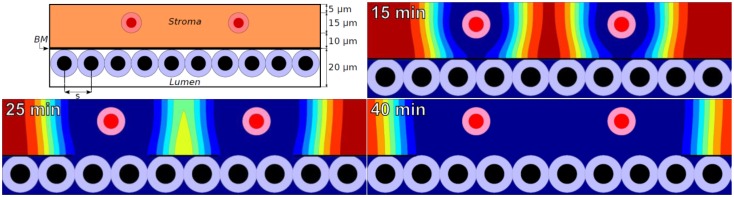
***Top left:*** initial configuration of epithelium (white lumen and tumor cells), a 100 nm basement membrane, stroma (orange), and stromal cells (red) that secrete MMPs. ***Remaining plots:*** ECM volume fraction [ranging from blue (0%) to red (85%)] at 15, 25, and 40 min using a widespread ECM-MMP model with a biophysically reasonable reaction-diffusion length scale (~10 μm) and degradation rate (~0.1–1 min^−1^).

We simulated MMP secretion by stromal cells, as one might expect in response to tumor-secreted pro-inflammatory signals. To simplify the analysis, we set *M* = 1 on the stromal cells and positioned them at a fixed 10 μm from the BM (Figure 1: top left).

In the simulations, MMPs etch out a “hole” in the ECM whose edge expands outward at ~1 μm per minute. See Figure 1 for the ECM distribution at 15, 25, and 40 min. This is consistent with an order of magnitude estimate using a Fisher–Kolmogorov-type traveling wave front speed:

$$\nu =2L\left(E\right)r_{M}\left(E\right)\sim 2\sqrt{D_{0}\left(1-E\right)Er_{M}}$$

gives speeds of 0–3.75 μm/min ~ 1 μm/min for 0 ≤ *E*  ≤ 0.90, where *L*(*E*) is as above and *r_M_*(*E*) = *r_M_* *E*.

A 1 μm/min expansion rate of the degraded region is comparable to experimentally measured motile tumor cell velocities [e.g., 58.56 ± 1.62 μm/h for neuN cells in (7)]. The predicted expansion speed is quantitatively consistent with localized ECM degradation “keeping pace” with motile cells as they “tunnel” through the ECM. The simple ECM-MMP model (with sufficient numerical resolution) can produce biologically reasonable results on small time and spatial scales.

However, if extrapolated over long times, this model predicts that a 10 cm diameter of tissue could be degraded in about a month! This outpaces typical tumor expansion rates by over an order of magnitude: brain tumors (among the fastest growing tumors) typically expand at 80–100 μm/day (18), requiring at least 500 days to infiltrate a 10 cm tissue. The simple MMP model would therefore predict an ever-widening gap between the advancing tumor front and the edge of the degraded tissue, contradicting typical observations that MMP activity is localized near the boundary of an advancing tumor.

This widely used model, once simulated accurately, does not adequately describe MMP-mediated tissue degradation around growing tumors. Neglected factors (e.g., activators and inhibitors) may be needed to confine proteolytic activity near tumor boundaries; similar approaches have been used to model urokinase-type plasminogen activators in tissue degradation (19). Alternatively, non-diffusing membrane-bound MMPs may be more relevant. New imaging technologies that dynamically capture ECM degradation could help select among possible alternative models (20, 21). Recent integrative experimental-computational work showed the critical role of MT-MMP activity during cancer cell invasion, finding that MT1-MMP turnover could be a potent anti-invasion therapeutic target (22). Ultimately, only carefully planned and executed experiments can help choose between these and other possible explanations.

### Assessing degradation of the basement membrane

A 100 nm BM cannot be properly resolved on a 1 μm mesh. Solving (1)-(2) by finite differences (with correct physical dimensions) requires a prohibitive 10 nm computational mesh. Some have investigated this problem by solving on non-physiological basement membranes [e.g., one cellular automaton mesh point, or 10 μm thick (10)], making it difficult to evaluate the models.

Let us instead analyze a simplified problem to estimate the time scale to degrade a BM. Consider a small piece of BM of cross-sectional area *A*, volume fraction *F*, and thickness *T*(*t*). The total amount of matrix *E*(*t*) in the BM section is *AF T*(*t*). If BM is degraded as in (2), then d*E*/d*t* = −*r_E_* *M*(*t*) *E*(*t*). If *M* is constant, then the time *t*_B_ required to degrade the BM to some threshold breaking amount *E*_B_ is given by

$$t_{B}=-1n\left(E\left(0\right)/E_{B}\right)/r_{B}M.$$

If *r*_E_ = 1/200 s^−1^ and *M* = 1, then a 100 nm section of BM is reduced to 10 nm thick [*E*_B_/*E*(0) = 0.1] in under 8 min, and to 1 nm thick (*E*_B_/*E*(0) = 0.01) in about 15 min.

This suggests several possibilities. (1) The cell “decision” of when to secrete MMPs is the limiting factor to penetrating the BM, rather than the proteolytic process itself. (2) Additional, non-modeled promoters/inhibitors are rate limiting. Only follow-up experiments can help determine the most plausible explanation, but rapid penetration of the BM by “willing” cells seems consistent with Boyden transwell migration assays (23) and known rapid (~minutes) transmigration of leukocytes through endothelial and epithelial layers and associated membranes (24, 25).

## Closing Thoughts

Accurate models are needed to simplify, analyze, and assess complex phenomena observed in cancer biology. In order to truly assess a model’s underlying assumptions, evaluate its predictive value, and study its potential clinical utility, one must use proper numerical methods, reasonable geometries, and experimental validation. As illustrated by the tissue degradation model above, neglecting any of these key factors can lead to inaccurate dynamics, and may potentially cause a team to prematurely accept biological hypotheses. Dynamic feedback between experimental and computational biology systems is necessary to drive and improve model development and refinement while ensuring that any resulting integrative platform is clinically relevant.

## Conflict of Interest Statement

The authors declare that the research was conducted in the absence of any commercial or financial relationships that could be construed as a potential conflict of interest.
